# Physicochemical property, antioxidant activity, and cytoprotective effect of the germinated soybean proteins

**DOI:** 10.1002/fsn3.822

**Published:** 2018-11-02

**Authors:** Chunxia Gao, Fengzhong Wang, Li Yuan, Junyi Liu, Dan Sun, Xuyan Li

**Affiliations:** ^1^ College of Food Engineering and Nutritional Science Shaanxi Normal University Xi'an China; ^2^ Institute of Food Science and Technology CAAS Beijing China

**Keywords:** antioxidant activity, apoptosis, benzo(a)pyrene, cytoprotective effect, germination soybean protein

## Abstract

Appropriate germination can improve the nutritional value and bioactivity of soybeans; however, few studies have assessed the effect of germination on soybean proteins. This study examined the physicochemical property, antioxidation, and cytoprotective effect of the germinated soybean proteins (Gsp). Gsp was extracted from soybeans which germinated for 0–3 days using the method of alkali‐solution and acid‐isolation extraction. The results showed that germination could digest soybean proteins into the smaller molecules; enhance the degree of hydrolysis, emulsifiability, and foaming capacity; increase the removal rate of ABTS, DPPH, $O_{2}^{-}$˙, and ˙OH radical; and decrease the reducing power and lipid peroxidation of Gsp. Additionally, Gsp was able to protect HL‐7702 human hepatocyte cells against benzo(a)pyrene (BaP)‐induced cytotoxicity through mediating the cell cycle arrest, suppressing apoptosis, and increasing reactive oxygen species (ROS) levels. This work demonstrated that germination could enhance the physicochemical property and antioxidant activity of Gsp, which also displayed the remarkable cytoprotective effect. This study provided a fundamental basis for substantiating dietary of Gsp used for resistance to oxidation and hepatic injury.

## 1 INTRODUCTION

Soybeans are rich in amino acids, nutrients, and bioactive components, and it has become a valuable dietary supplement to promote health, particularly in rural areas among Asian countries where seasonal fruits and vegetables are unavailable all year‐round (Krishnan et al., 2015). As a nutritionally excellent food, more than 500,000 tons of soybean sprouts are consumed annually in Korea, Japan, and China (Lee, Shannon, Jeong, Lee, & Hwang, 2007). There are 40% of proteins in soybeans, which is the most inexpensive source of high‐nutritional‐quality protein and the world's predominant commercially available vegetable protein. It is well known that soybean protein has the remarkable antioxidant, anti‐inflammatory, and anticancer properties (Lee et al., 2014; Vernaza, Dia, & Mejiab, 2012; Yuan et al., 2015).

As a simple low‐cost process, germination is an effective technology for improving the nutritional and functional qualities of soybean: (a) the degradation of the antinutritional factors and trypsin inhibitor (Bau, Villaume, Nicolas, & Méjean, 2015; Harini, Basetty, Jasti, Lavanya, & Fadnavis, 2017); (b) the accumulation of bioactive phytochemicals, including vitamins, phytosterols, and tocopherols (Kaushik, Satya, & Naik, 2010); (c) the enhancement of isoflavone content (Chen & Chang, 2015); and (d) oligosaccharides hydrolysis (raffinose and stachyose; Félix et al., 2013). Moreover, the content and digestibility of soluble proteins are also improved during germination process (Bau et al., 2015). However, only a few studies have been devoted to assess the effect of germination on the physicochemical property, antioxidant, and cytoprotective effect of soy proteins.

The present study demonstrated the physicochemical properties and antioxidant activity of Gsp through examining sulfhydryl, disulfide bonds, amino acid composition, degree of hydrolysis, oil and water absorption capacity, emulsifiability, and foaming capacity, and assessing radical scavenging activity (DPPH, ABTS, $O_{2}^{-}$˙, and ˙OH), reducing power, and inhibition rate of lipid peroxidation. Additionally, the cytoprotective effect of Gsp on HL‐7702 cells exposed in food injurant BaP was also examined. This study provided a fundamental basis for substantiating dietary of Gsp used for resistance to oxidation and hepatic injury.

## 2 MATERIALS AND METHODS

### 2.1 Chemicals and reagents

The soybeans (Qindou 8#, 85(6)‐5‐1‐1) of different germination stage were purchased from a local market in Shaanxi Province of China. MTT (purity ≥98%) and benzo[α]pyrene (purity ≥96%) were obtained from Sigma (St. Louis, MO, USA). DCFH‐DA was obtained from BestBio Co. (Shanghai, China). BCA protein kit and other cell culture reagents were purchased from Thermo Fisher (Shanghai, China). All of the other chemicals were made in China and were of the highest available grade.

### 2.2 Germinated soy proteins extraction

#### 2.2.1 Preparation of non‐germinated and germinated soybean flour

**Figure 1:**
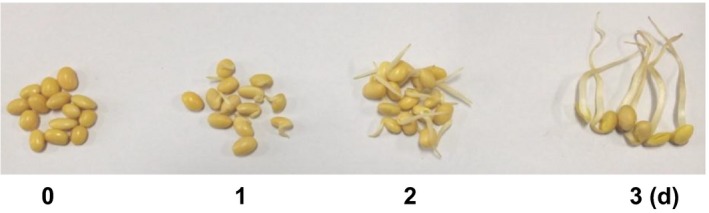
Appearances of yellow soybean sprout. The first one is the control soybean, and the 1st, 2nd, and 3rd are the soybean sprouts germinated for 1, 2 and 3 days, respectively

The same batch of mature and full soybean grain which was germinated for 0, 1, 2, and 3 days, respectively, and the corresponding length of soybean sprouts was 0, 0.5–1, 1–3, and 3–5 cm (Figure 1). Soybean sprouts were dried at 40°C in the electricity heat drum wind drying oven to constant weight (about 10 hr; GZX‐9146 MBE, Shanghai Boxun Medical Biological Instrument Corp., Shanghai, China) and grounded into fine powder (60 mesh) by using an IKA^®^ all basic mill (IKA Works Inc., Wilmington, USA) for 3 min. Then, the soybean flour obtained from the above method was used to protein extraction by the method of alkali extraction and acid precipitation (Robles‐Ramírez, Ramón‐Gallegos, Reyes‐Duarte, & Mora‐Escobedo, 2012). Briefly, the soybean flour was defatted with hexane and suspended in distilled water (1:20), and pH was adjusted to 9.0 with 1 mol/L NaOH. The extraction was stirred for 45 min at room temperature and centrifuged at 10,000 *g* for 30 min at 4°C. The pH of supernatant was adjusted to 4.5 with 1 mol/L HCl and the precipitate collected by centrifugation at 10,000 *g* for 30 min at 4°C, followed by freeze‐drying and stored at 4°C.

### 2.3 Determination of physicochemical property

#### 2.3.1 SDS‐PAGE electrophoresis of Gsp

The protein concentration of Gsp was determined using BCA kit. Protein samples were diluted in Laemmli buffer with 5% β‐mercaptoethanol and then boiled for 5 min prior to loading. Finally, Gsp were separated by SDS‐PAGE and exposed using a Molecular Imager ChemiDoc 510 system (UVP Corporation, USA).

#### 2.3.2 Sulfhydryl and disulfide bond contents measurement

Sulfhydryl (SH) and disulfide bond (S‐S) contents of Gsp were measured according to the method of Jia, Huang, and Xiong (2016). SH and S‐S were determined by the Equations (1) and (2) as follows:

(1) $$\text{SH}\;\left(\mu M/g\;\text{protein}\right)\;=\;73.53\;\times \;A_{412}/C$$

(2) $$\text{S‐S}\;\left(\mu M/g\;\text{protein}\right)\;=\;\left({\text{SH}}_{T}\;-\;{\text{SH}}_{F}\right)/2$$

where A_412_ is the absorbance at 412 nm, C is the protein concentration (mg/ml), and 73.53 is derived from 10^6^/13,600 (13,600 is Ellman's reagent molar absorptivity). SH_T_ is the free sulfhydryl group. SH_F_ is the total sulfhydryl group.

#### 2.3.3 Determinations of degree of hydrolysis

The degree of hydrolysis (DH) of Gsp was determined using the modified OPA method described by Nielsen, Petersen, and Dambmann (2010). DH% was calculated using the following equations (Equation (3), (4), (5)).

(3) $$\begin{array}{cc} {\text{Serine‐NH}}_{2}\;=\; & \left({\text{OD}}_{\mathrm{sample}}-{\text{OD}}_{\mathrm{blank}}\right)/\left({\text{OD}}_{\mathrm{standard}}-{\text{OD}}_{\mathrm{blank}}\right)\\ & \;\times \;0.9516\;\text{meqv}/L\;\times \;0.1\;\times \;100/(X\;\times \;P) \end{array}$$

(4) $$h\;=\;({\text{Serine‐NH}}_{2}-\beta )/\alpha$$

(5) $$\text{DH}\;=\;h/h_{\mathrm{tot}}\;\times \;100\%$$

where *X* = g sample, *P* = protein % in sample, 0.1 is the sample volume in liter (L), *h* = number of hydrolyzed bonds (meqv/g protein), *h*_tot_ = total number of peptide bonds per protein equivalent (7.8 specific to soy protein), *β* = 0.342 (specific for soy protein), and *α* = 0.970 (specific for soy protein).

#### 2.3.4 Water and oil holding capacities

Water and oil absorption capacities were determined according to the method of Zhang, Yang, Tang, Chen, and You (2015). 2 g of samples was mixed with 20 ml distilled water or corn oil (Sigma) in 50‐ml centrifuge tubes. Each mixture was shocked for 1 min, allowed to stand for 30 min, and then centrifuged at 2,000 × *g* for 30 min. The results were expressed as ml of liquid retained per g of sample.

#### 2.3.5 Protein solubility at different pH levels

Protein solubility was determined by dispersing samples in distilled water to obtain a final solution of 0.2% (w/w) in protein. The pH values of protein solution were adjusted from 8 to 3 and then were centrifuged at 10,800 × *g* for 30 min. The content of protein in the resulting solution was analyzed using BCA protein kit.

#### 2.3.6 Analysis of amino acids

Samples were acid hydrolyzed at 110°C for 24 hr in 6 M HCl in vacuum‐sealed tubes. Amino acids levels were determined by an automatic amino acid analyzer (L‐8900, Hitachi, Ltd., Japan).

#### 2.3.7 Emulsifiability and emulsifying stability

The emulsifying activity index (EAI) and emulsion stability index (ESI) of Gsp were measured following the method by Pearce and Kinsella (1978) and were calculated using the following equations (Equation (6) and (7)):

(6) $$\text{EAI}\;\left(m^{2}/g\right)\;=\;V_{1}/V_{0}\;\times \;100\%$$

(7) $$\text{ESI}\;\left(\%\right)\;=\;V_{2}/V_{0}\;\times \;100\%$$

where *V*_0_ refers to the total volume of Gsp solution samples, and *V*_1_ and *V*_2_ are the volume of emulsion layer and the volume of emulsion layer after standing.

#### 2.3.8 Foaming capacity and foam stability

The method of Zhang, Zhang, Wang, and Guo (2012) was used to evaluate foaming capacity (FC) and foaming stability (FS) of Gsp. They were calculated using the following equations (Equation (8) and (9)):

(8) $$\text{FC}\;\left(\%\right)\;=\;\left(V_{1}-V_{0}\right)/V_{0}\;\times \;100$$

(9) $$\text{FS}\;\left(\%\right)\;=\;\left(V_{2}-V_{0}\right)/V_{1}\;\times \;100$$

where *V*_0_ refers to the volume before whipping, *V*_1_ is the volume after whipping, and *V*_2_ is the volume after standing.

### 2.4 Determination of antioxidant activity

#### 2.4.1 DPPH radical scavenging assay

DPPH radical scavenging activity of Gsp was evaluated according to the method reported by Alam, Bristi, and Rafiquzzaman ((1)) and calculated using the following equation (Equation (10)):

(10) $$\text{DPPH radical scavenging activity}\;\left(\%\right)\;=\;\left(1\;-\;A_{1}/A_{0}\right)\;\times \;100$$

where A_0_ refers to the absorbance without Gsp, and A_1_ is the absorbance in the presence of Gsp. EC_50_ value (mg/ml) obtained by interpolation from linear regression analysis represents the effective concentration at which DPPH radicals were scavenged by 50%.

#### 2.4.2 ABTS radical scavenging assay

ABTS radical scavenging activity was measured using the method developed by Re et al. (1999). It was calculated as follows (Equation (11)):

(11) $$\text{ABTS radical scavenging activity}\;\left(\%\right)\;=\;\left(1-A_{1}/A_{0}\right)\;\times \;100$$

where A_0_ refers to the absorbance without Gsp, and A_1_ is the absorbance in the presence of Gsp. EC_50_ value (mg/ml) obtained by interpolation from linear regression analysis represents the effective concentration at which ABTS radicals were scavenged by 50%.

#### 2.4.3 $O_{2}^{-}$˙ scavenging activity

$O_{2}^{-}$˙ scavenging activity was measured based on the described method (Bajpai, Baek, & Kang, 2017) and calculated using the following equation (Equation (12)):

(12) $$O_{2}^{-\cdot }\;\text{scavenging activity}\;\left(\%\right)\;=\;\left[\left(A_{\mathrm{control}}\;-\;A_{\mathrm{sample}}\right)/A_{\mathrm{control}}\right]\;\times \;100$$

where A_control_ was the absorbance without sample, and A_sample_ was the absorbance with sample. EC_50_ value (mg/ml) obtained by interpolation from linear regression analysis represents the effective concentration at which $O_{2}^{-}$˙ radicals were scavenged by 50%.

#### 2.4.4 ˙OH scavenging activity

˙OH radical generated by the Fe^3+^/ascorbic acid system has been studied by the method of Stamenic et al. (2014), and it was calculated as follows (Equation (13)):

(13) $$\text{DPPH radical scavenging activity}\;\left(\%\right)=\left(1-A_{1}/A_{0}\right)\times 100$$

where A_0_ is the absorbance of the control sample, and A_1_ is the absorbance of sample. The EC_50_ value (mg/ml) obtained by interpolation from linear regression analysis represents the effective concentration at which ˙OH radicals were scavenged by 50%.

#### 2.4.5 Reducing power assay

The reducing power of Gsp was determined using the ferric reducing ability of plasma assay (Almansoub, Asmawi, & Murugaiyah, 2014). A standard curve was prepared using different concentrations (10–100 mmol/L) of FeSO_4_. Reduction percentage was calculated as follows (Equation (14)):

(14) Percentage reduction activity=[1−(1−As/Ac)]×100

where As is the absorbance of the sample, and Ac is the absorbance of the standard at maximum concentration. EC_50_ value (mg extract per ml) obtained from the linear regression analysis represents the effective concentration at which the absorbance is 0.5 at 593 nm.

#### 2.4.6 Determination of lipid peroxidation

Malondialdehyde (MDA) is one of the end products of lip peroxidation, and it was determined according to Szőllősi, Varga, Erdei, and Mihalik (2009). Gsp was mixed with 10% (w/v) trichloroacetic acid and 0.5% (w/v) thiobarbituric acid, heated for 15 min at 96°C, then cooled down to room temperature, and centrifuged at 3,600 × *g* for 5 min. Absorbance of the supernatant was read at 532 nm and 600 nm, respectively. The value of MDA was expressed as μmol/g.

#### 2.4.7 Antioxidant activity of Gsp after digestion in vitro

Antioxidant activity of Gsp after digestion in vitro was measured according to the method proposed by Shim et al. (2012). Briefly, pepsin and trypsin reacted with proteins at 37°C water bath for 4 hr, respectively, adjusted pH to 7.0. Hydrolyzate was used for the determination of antioxidant capacity (DPPH, ABTS, and ˙OH) through the above method.

### 2.5 Determination of cytoprotective effect

#### 2.5.1 Cell culture

HL‐7702 human hepatocyte cells were obtained from Kunming Institute of Zoology, Chinese Academy of Science, and were cultured in RPMI‐1640 medium with 10% FBS and 1% penicillin‐streptomycin at 37°C in a humidified incubator (5% CO_2_, 95% air).

#### 2.5.2 Cell viability

Cells were seeded in 96‐well plates at a density of 1 × 10^6^ cells/ml. After 12 hr, cells were treated with different concentrations of Gsp (0, 5, 10, 25, 50, 100, and 125 mg/L) or 63 mg/L BaP for 24 hr. Subsequently, 100 μl of 0.5% (w/v) MTT was added to each well and incubated for 4 hr. Then, removed MTT and added DMSO to dissolve the formazan crystals. Absorbance at 490 nm was measured with a microplate reader (Thermo Fisher, USA).

#### 2.5.3 Cell cycle analysis

Cell cycle was analyzed using flow cytometry. After treatment, the detached cells in the medium were collected with PBS and combined with the remaining adherent cells. After centrifugation, cell pellets were resuspended and fixed with 70% ethanol at 4°C overnight, and then resuspended in 1.0 mg/ml RNase (Sigma). Subsequently, 50 μg/ml propidium iodide (PI, Sigma) stain solution was added, incubated in the dark for 30 min at room temperature, and finally analyzed by the GUAVA^®^ easyCyte™ 8HT flow cytometry (Millipore Corporation, USA).

#### 2.5.4 Assessment of cell apoptosis

Early and late apoptotic cells were detected using an Annexin V‐FITC/PI Apoptosis Detection Kit (BestBio, China) following the manufacturer's instruction. Briefly, cells were suspended in binding buffer (adding 5 μl of annexin V‐FITC and 10 μl of PI). Thereafter, samples were incubated in the dark for 10 min at 4°C and then analyzed on the flow cytometer (BD Facscalibur, USA).

#### 2.5.5 Measurement of ROS

Reactive oxygen species (ROS) was measured using DCFH‐DA probes. After treatment, cells were incubated with 5 μmol/L of DCFH‐DA at 37°C with 5% (v/v) CO_2_ for 30 min. Subsequently, cells were washed three times with serum‐free medium, and the fluorescence intensity in cells was determined using fluorescence microscope (Olympus Optical Co., Ltd., Japan) and flow cytometry, respectively.

### 2.6 Statistical analysis

All experiments were performed three times, and data are presented as the means ± standard errors (*SE*). Significant differences between measurements for the control and treated samples were analyzed using one‐way factorial analysis of variance and Duncan's post hoc test with SPSS 16.0.

## 3 RESULTS

### 3.1 Effect of germination on the chemical composition of soybean protein

The secondary structure and chemical composition of Gsp were changed by germination. As shown in Table 1, the soluble protein content of Gsp in 0, 1, 2, and 3 days germination was 0.22, 0.27, 0.29, and 0.30 mg/g, respectively, showing that germination could significantly increase the protein content of soybean. SH content was significantly (*p* ≤ 0.05) lower at 1d than that in 0d, as well as S‐S content. As the continuation of germination, DH and water absorption capacity of Gsp increased gradually, while oil absorption capacity appeared declined. For the solubility of Gsp in different pH values, the lowest solubility was at pH 7.0, and the highest solubility was at pH 3.0.

**Table 1:** Physicochemical properties of germinated soybean proteins

	Germinating time (day)			
Index	0	1	2	3
Soluble protein content (mg/g)	0.22 ± 0.01^b^	0.27 ± 0.01^a^	0.29 ± 0.01^a^	0.30 ± 0.01^a^
Crude protein content (%)	35.47 ± 0.82^a^	33.05 ± 0.78^b^	32.78 ± 0.62^b^	31.49 ± 0.55^b^
Sulfhydryl content (μM/g)	1.60 ± 0.08^a^	1.36 ± 0.12^b^	1.18 ± 0.05^b^	1.35 ± 0.05^b^
Disulfide bond content (μM/g)	7.81 ± 0.16^a^	5.98 ± 0.06^c^	6.91 ± 0.07^b^	7.05 ± 0.04^b^
Degree of hydrolysis (%)	2.28 ± 0.01^a^	2.23 ± 0.05^a^	2.29 ± 0.16^a^	32.39 ± 0.27^a^
Water absorption capacity (g/L)	1.46 ± 0.04^bc^	1.43 ± 0.02^c^	1.52 ± 0.01^b^	1.77 ± 0.01^a^
Oil absorption capacity (g/L)	3.40 ± 0.10^a^	3.10 ± 0.03^b^	2.23 ± 0.10^b^	2.91 ± 0.01^b^
Solubility^a^ (%)				
pH 7.0	13.55 ± 0.89^a^	16.29 ± 2.67^a^	14.92 ± 0.69^a^	16.8 ± 1.18^a^
pH 8.0	22.95 ± 0.42^b^	21.41 ± 0.21^b^	24.55 ± 0.85^a^	28.16 ± 1.10^a^
pH 3.0	93.67 ± 0.42^b^	97.07 ± 0.68^b^	98.97 ± 0.52^a^	99.46 ± 1.93^a^

*Notes*

Values shown are mean ± *SD*,* n* = 9. The means in the row not sharing a common letter (^abc^) are significantly different among groups (*p *≤* *0.05).

a Solubility determined in DW (deionized water at pH 7.0), 0.05 M Tris‐HCl buffer (pH 8.0), or 0.05 M Gly‐HCl (pH 3.0).

Table 2 presents the composition of amino acid of Gsp. Asp and Glu showed a trend of increasing with the germination times. Met showed a significant (*p* ≤ 0.05) increase from days 0 to 3 of germination, and due to the restrictions in experimental conditions, Cys was not detected in this study. Additionally, Tyr firstly increased and then decreased during the germination, and reached the highest value at Day 2. Generally speaking, total amino acid (TAA), total essential amino acid (EAA), and no essential amino acid (NEAA) tended to be on the rise.

**Table 2:** Composition of amino acid in the germinated soybean proteins (mg/g.pr)

Amino acids	Abbreviation	Germinating time (day)			
		0	1	2	3
Aspartic acid	Asp	83.233	83.015	80.500	90.284
Threonine^a^	Thr	28.425	28.466	27.579	29.429
Serine	Ser	39.490	39.721	38.191	41.417
Glutamic acid	Glu	152.216	145.761	142.300	149.882
Glycine	Gly	29.273	28.458	26.955	30.401
Alanine	Ala	43.092	47.882	49.638	45.246
Valine^a^	Val	32.576	35.811	36.974	36.421
Methionine^a^	Met	9.318	11.186	12.980	16.618
Isoleucine^a^	Ile	25.590	25.107	24.021	33.716
Leucine^a^	Leu	52.927	52.838	50.414	62.288
Tyrosine^b^	Tyr	35.154	40.747	43.546	38.845
Phenylalanine^a^	Phe	38.638	43.143	45.193	37.202
Lysine^a^	Lys	45.037	45.266	45.452	46.216
Arginine	Arg	57.820	53.804	51.791	55.584
Histidine^a^	His	20.604	21.308	21.908	22.220
Proline	Pro	37.382	34.330	33.042	38.187
Cysteine^b^	Cys	–	–	–	–
Tryptophan^a^	TRP	–	–	–	–
Free Ammonia	NH3	7.625	7.656	7.958	8.326
Lys/Arg		0.779	0.841	0.878	0.831
TAA		730.775	736.843	730.484	773.956
EAA		288.269	303.872	308.067	322.955
EAA/TAA		0.394	0.412	0.421	0.417
EAA/NEAA		0.651	0.702	0.729	0.716

*Notes*

Values shown are mean ± *SD*,* n* = 9.

EAA: total essential amino acid; NEAA: no essential amino acid; TAA: total amino acid; –: not detected.

^a^Essential amino acid. ^b^Conditionally essential amino acid.

### 3.2 SDS‐PAGE of Gsp

**Figure 2:**
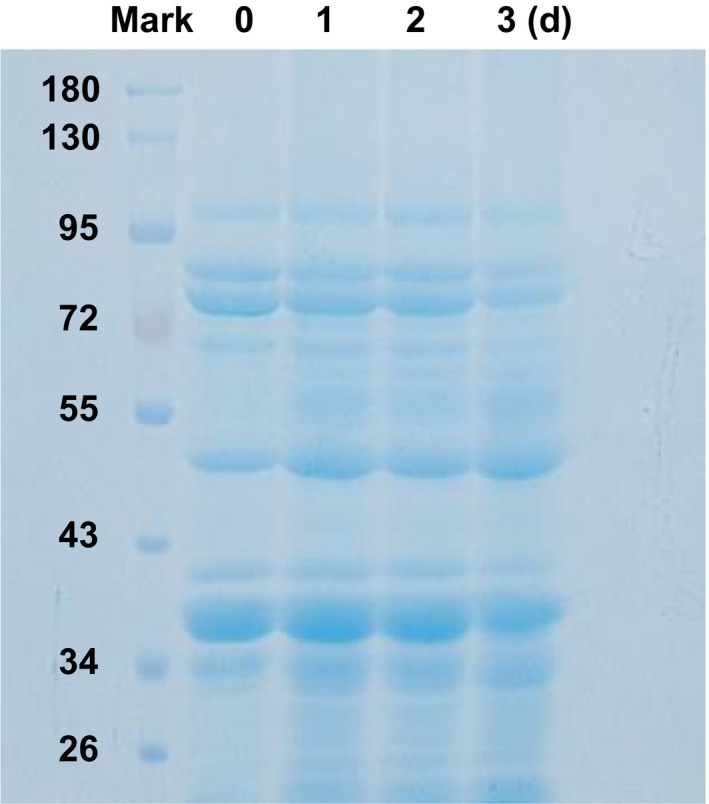
SDS‐PAGE of Gsp. M means molecular mass markers; Lane 1, no germination; Lane 2, 1 d of germination; Lane 3, 2‐d germination; and Lane 4, 3‐d germination

As shown in Figure 2, the small molecular weight proteins in control group were slightly lighter than that in the 3‐d germinated samples. During germination, the soybean proteins were digested into the smaller molecules proteins which distributed in 34–43, 43–55, and 72–95 kD.

### 3.3 Functionality properties of Gsp

**Figure 3:**
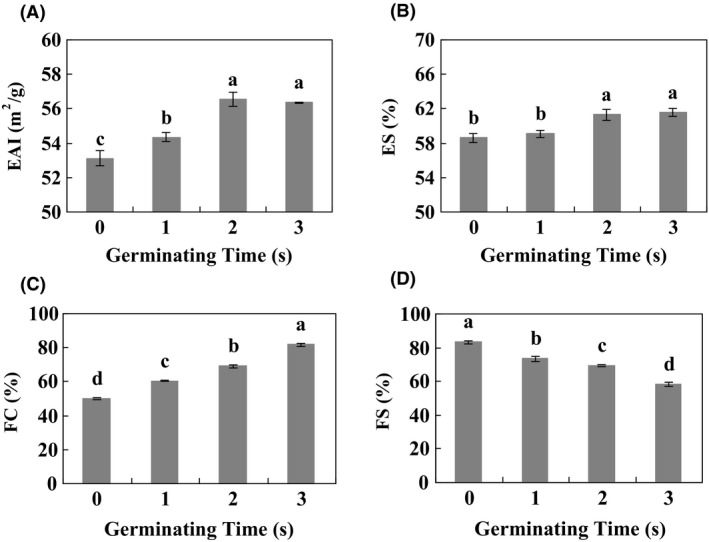
Functionality properties of Gsp. Emulsifying activity index (A), emulsifying stability (B), foaming capacity (C), and foaming stability (D) of Gsp. Values are expressed as means ± *SD* of nine samples in each group. Different lower‐case letters (a, b, c, and d) indicate significant difference for the different germinated soybean protein samples at *p* ≤ 0.05

Emulsifying activity index (EAI) and emulsifying stability index (ESI) of Gsp were notably (*p *≤* *0.05) increased by germination, while no significant differences were been found between 2‐d and 3‐d germination (Figure 3A,B). Besides, foaming capacity (FC) showed a gradual and significant (*p *≤* *0.05) increase, and foaming stability (FS) showed a contrary tendency (Figure 3C,D). All these results showed that germination was able to enhance the functionality properties of soybean proteins.

### 3.4 Antioxidant activities of Gsp

**Figure 4:**
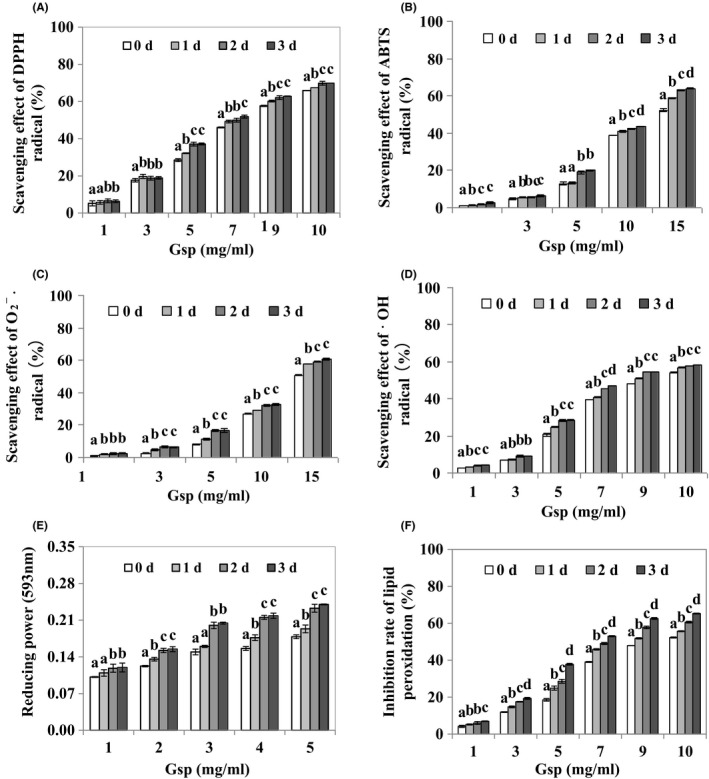
Antioxidant activity of Gsp. Scavenging effect of DPPH (A), ABTS (B), $O_{2}^{-}$˙ (C), ˙OH radical (D), reducing power (E), and inhibition rate of lipid peroxidation (F) of Gsp. Each value is expressed as a mean ± *SD*, and lower‐case letters (a, b, c, and d) indicate significant difference for the different germinated soybean protein samples at *p* ≤ 0.05

As shown in Figure 4, with the increase in germination time, the removal rate of ABTS, DPPH, $O_{2}^{-}$˙, and ˙OH radical, decrease in the reducing power, and inhibition rate of lipid peroxidation of Gsp showed a gradual and significant (*p *≤* *0.05) increment trend. Table 3 shows that compared to 0d, EC_50_ of DPPH, ABTS, $O_{2}^{-}$˙, ˙OH radical, and lipid peroxidation of Gsp germinated for 3d decreased by 17.87%, 21.85%, 12.75%, 11.00%, and 27.25%, respectively (Table 3). Generally speaking, Gsp had the significant antioxidant activities in a dose‐dependent manner, and germination could enhance antioxidant activities of soybean protein.

**Table 3:** EC_50_ values in the DPPH, ABTS, $O_{2}^{-}$˙, ˙OH radical, and lipid peroxidation of germinated soybean proteins

Germinating time (day)	EC_50_ (mg/ml)				
	DPPH	ABTS	$O_{2}^{-}$˙	˙OH	Lipid peroxidation
0	7.87 ± 0.11^a^	14.19 ± 0.08^a^	14.98 ± 0.13^a^	9.27 ± 0.11^a^	9.43 ± 0.04^a^
1	7.41 ± 0.13^b^	12.87 ± 0.07^b^	13.67 ± 0.12^b^	8.88 ± 0.07^b^	8.72 ± 0.06^b^
2	7.04 ± 0.11^c^	11.99 ± 0.08^c^	13.43 ± 0.08^c^	8.40 ± 0.12^c^	7.86 ± 0.07^c^
3	6.39 ± 0.20^c^	11.09 ± 0.09^d^	13.07 ± 0.12^c^	8.25 ± 0.05^c^	6.86 ± 0.11^d^

*Note*

Values shown are mean ± *SD*,* n* = 9. The means in the row not sharing a common letter (^abc^) are significantly different among groups (*p *≤* *0.05).

**Figure 5:**
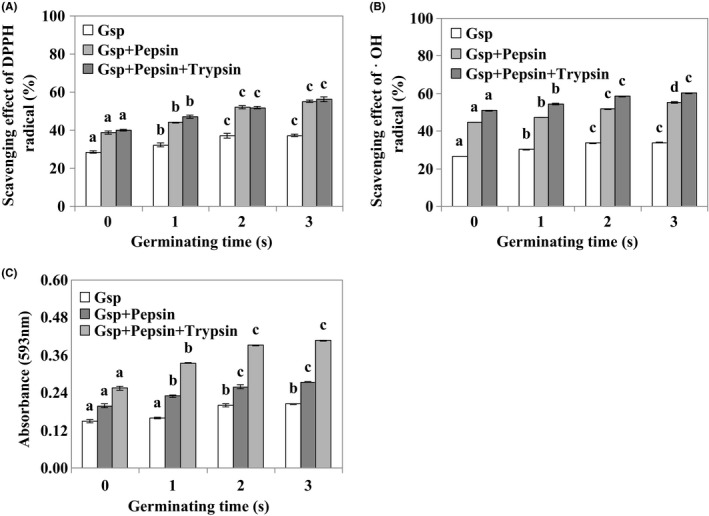
Antioxidant activity of Gsp after digestion in vitro. Scavenging effect of DPPH (A) and ˙OH radical (B), and reducing power (C). Each value is expressed as a mean ± *SD*, and lower‐case letters (a, b, and c) indicate significant difference for the different germinated soybean protein samples at *p* ≤ 0.05

Additionally, we also access the antioxidant activities of Gsp after digestion in vitro. After digestion with pepsin and trypsin, Gsp also had a high clearance rate for DPPH, ˙OH, and reducing power (Figure 5), suggesting that Gsp possessed notable antioxidant activities.

### 3.5 Effect of Gsp on BaP‐induced injury in HL‐7702 cells

**Figure 6:**
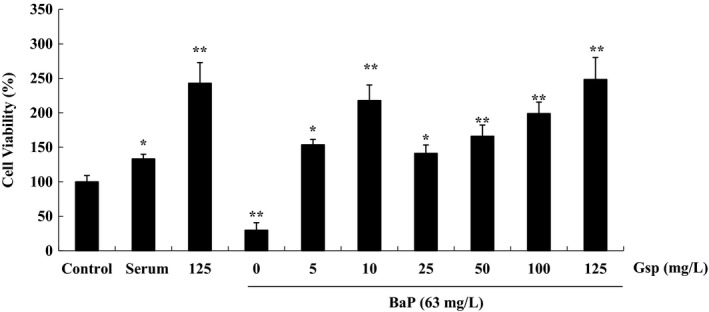
Effect of Gsp on BaP‐induced injury in HL‐7702 cells. Cell viability was determined by MTT. Each bar represents the mean ± *SD* of nine independent experiments. * *p* ≤ 0.05 and ** *p* ≤ 0.01 indicate statistically significant differences versus control group

A significant (*p *≤* *0.01) cytoprotective effect of Gsp on HL‐7702 cells induced by BaP was observed at 63 mg/L, compared to the untreated cells (Figure 6). It was found that cells viability notably (*p *≤* *0.01) increased with Gsp concentrations, indicating that Gsp could protect liver cells from BaP‐induced injury.

### 3.6 Effects of Gsp on BaP‐induced cell cycle arrest in HL‐7702 cells

**Figure 7:**
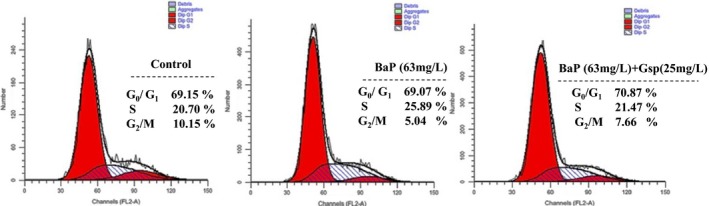
Effects of Gsp on BaP‐induced cell cycle arrest in HL‐7702 cells. Cell Cycle was measured using flow cytometry. Horizontal and vertical axes indicate the relative nuclear DNA content and number of cells, respectively

As shown in Figure 7, a strong S‐phase arrest in HL‐7702 cells was been found after BaP treatment. Compared with BaP group, the treatment with BaP (63 mg/L) plus Gsp (25 mg/L) led to a significant (*p* ≤ 0.01) decrease in cell population of S phase from 25.89% to 21.47%, and the cell population of G2/M phase increased from 5.04% to 7.66%. Meanwhile, cell population slight accumulation was observed on the G0/G1 phase in the tested cell lines. The results suggest that Gsp weakened cell cycle arrest ability of BaP on HL7702 cells.

### 3.7 Effects of Gsp on BaP‐induced apoptosis in HL‐7702 cells

**Figure 8:**
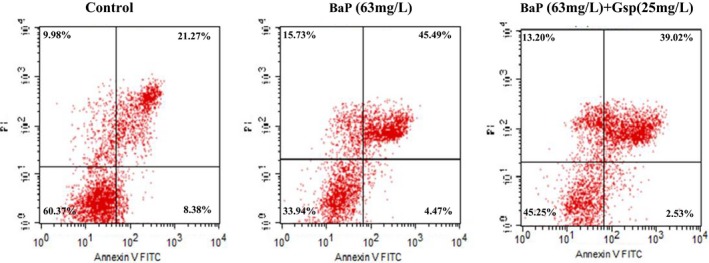
Effects of Gsp on BaP‐induced apoptosis in HL‐7702 cells. After treatment, cells were double‐stained with annexin V‐FITC and PI, and then cells were analyzed by flow cytometry

As shown in Figure 8, BaP resulted in about 4.47% of the cells going into early apoptotic phase, and 45.49% of cells was going into late apoptotic phase. However, both the early and late apoptotic cells were significantly decreased by Gsp treatment. Taken together, these results revealed that the pretreatment with Gsp could protect HL‐7702 cell from BaP‐induced apoptosis.

### 3.8 Effects of Gsp on ROS in BaP‐treated cells

**Figure 9:**
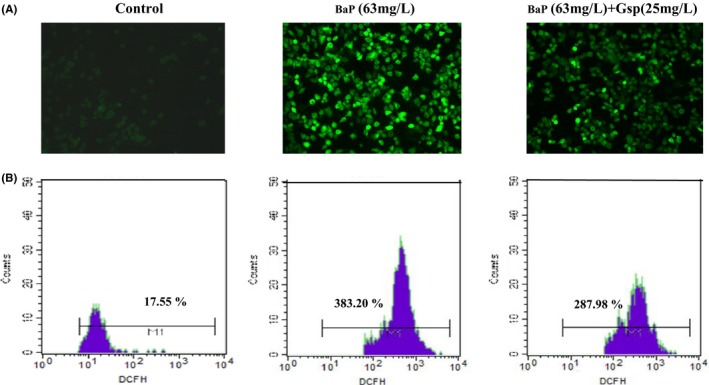
Effects of Gsp on reactive oxygen species (ROS) in BaP‐treated HL‐7702 cells. ROS levels were measured by fluorescent microscope (A) and flow cytometry (B) after incubation with DCFH‐DA. Scale bar = 100 μm

Accumulating evidence indicates that intracellular ROS can trigger apoptosis, we next determined whether Gsp protected HL‐7702 cell from BaP‐induced apoptosis through decreasing the generation of ROS. As shown in Figure 9, cells exposed to BaP displayed a significant increase in fluorescence intensity signals, compared with control group. Gsp markedly inhibited the generation of intracellular ROS induced by BaP, indicating that Gsp could inhibit apoptosis induced by BaP through decreasing intracellular ROS levels.

## 4 DISCUSSION

Soybean has various biological functions; however, the effects of germination on the physicochemical properties, antioxidant activity, and cytoprotective properties of soy protein remain unclear. The present study found that germination could not only change secondary structure of soybean proteins, but also promote physicochemical properties and antioxidant activity. In addition, Gsp could effectively inhibit cell damage induced by BaP. Gsp percentage decreased slightly as the germination time goes on (Table 1); it may be because some protein was metabolized during germination.

It has reported that germination can change the secondary structure of soybean proteins, and protein was digested into smaller molecules and reused for new protein synthesis for plant growth during germination (Chen & Chang, 2015). Solubility and the degree of hydrolysis are one of the most important characteristics of proteins. Solubility decreases at pH around proteinʼs PI. Consistent with these previous studies, our results found that germination could significantly (*p* ≤ 0.05) enhance degree of solubility and hydrolysis of Gsp through digesting into smaller molecules during germination time (Table 1, Figure 3). Amino acids are very rich in soybean. The present study observed that germination further improved the amino acids levels of soybean, except a conditionally essential amino acid Cys (Table 2). Lys content was slightly increased after germination, and no significant effect was found in Asp and Glu.

The functionality properties (EAI and FC) of protein are very important for the practical performance as emulsifying agents. Emulsifier is used to stabilize the emulsion depends on the oil–water interfacial area, and the emulsifying capacity also depends on the size of the droplets during agitation (Ritzoulis et al., 2014). In food systems, foams are often very complex, including several phases such as a mixture of gases, subdivided solids, subdivided liquids, multicomponent solutions of water, polymers, and surfactants (Richert, 1979). The high FC and low FS of Gsp were observed in the present investigation (Figure 3), and it may have been due to the formation of stable molecular layers in the air–water interface, which impart texture, stability, and elasticity of foams.

Antioxidant capacity is the most extensively investigated bioactivity in germinated edible seeds and sprouts, and it has reported that germination can change the antioxidant capacity in many edible seeds, such as wheat, rice bean, and so on (Chen et al., 2017; Sritongtae, Sangsukiam, Morgan, & Duangmal, 2017). It has reported that germination process can increase soluble protein concentration and resulted in the formation of bioactive compounds (free amino acids and oligopeptides; Huang, Cai, & Xu, 2014; Martinez‐Villaluenga et al., 2010). However, effect of germination on antioxidant capacity of soy proteins remains unknown. In our study, the result found that Gsp effectively eliminated DPPH, ABTS, $O_{2}^{-}$˙, and ˙OH free radicals, and decreasing reducing power and lipid peroxidation (Figure 4), showing that germination is able to strengthen antioxidant activity of soy proteins.

Additionally, to examine whether antioxidant capacity of Gsp will decrease or disappear in body, we simulated the digestive reaction in vitro. The results showed that, after digestion with pepsin and trypsin, Gsp also had a high clearance rate for DPPH, ˙OH, and reducing power (Figure 5), and it was in agreement with a report that Indian bean proteins digested by pepsin and trypsin also showed a high clearance rate for different radicals (Vadde, Pochana, & Pillatla, 2010). All the results suggest that Gsp possessed the notable antioxidant activity.

BaP is universally acknowledged to be a cancerogen. It is widely presented in a large number of high‐temperature‐processed foods and leads to the severe organism injury. It is reported that plant flavonoids and polyphenols can prevent from BaP damage (Kasala et al., 2016; Omidian, Rafiei, & Bandy, 2017; Sreelatha, Jeyachitra, & Padma, 2011); however, there were few reports about whether Gsp could protect cells from BaP‐induced damage. Our study found that Gsp could protect HL‐7702 cells from BaP through increasing cell viability, relieving cell cycle arrest, inhibiting apoptosis, and reducing ROS levels (Figure 6, 7, 8, 9), indicating a significant hepatoprotective effect.

## 5 CONCLUSION

The present work demonstrated that germination could digest soybean proteins into the smaller molecules and enhance functionality properties and antioxidant activity of soy proteins. Additionally, Gsp could protect cells from damage induced by BaP through increasing cell viability, inhibiting cell cycle arrest and apoptosis, and reducing ROS levels, showing that Gsp could be used for resistance to oxidation and hepatic injury, as a functional foods and dietary supplements. However, we only primarily assessed the cytoprotective properties of Gsp in this paper, the possible underlying mechanisms required to research in further studies.

## CONFLICT OF INTEREST

The authors declare that there are no conflict of interests.

## ETHICAL STATEMENT

This study does not involve any human or animal testing.
